# An evaluation of the emerging vaccines against influenza in children

**DOI:** 10.1186/1471-2458-13-S3-S14

**Published:** 2013-09-17

**Authors:** Harish Nair, Eva Shi May Lau, W Abdullah Brooks, Ang Choon Seong, Evropi Theodoratou, Lina Zgaga, Tanvir Huda, Suresh S Jadhav, Igor Rudan, Harry Campbell

**Affiliations:** 1 Centre for Population Health Sciences, Global Health Academy, The University of Edinburgh, UK; 2 Public Health Foundation of India, New Delhi, India; 3 International Centre for Diarrhoeal Disease Research, Bangladesh (ICDDR,B), Dhaka, Bangladesh; 4 Department of International Health, Bloomberg School of Public Health, Johns Hopkins University, Baltimore, MD, USA; 5 Serum Institute of India Limited, Pune, India

## Abstract

**Background:**

Influenza is an under-appreciated cause of acute lower respiratory infections (ALRI) in children. It is estimated to cause approximately 20 million new episodes of ALRI in children annually, 97% of these occurring in developing countries. It is also estimated to result in 28000 to 112000 deaths annually in young children. Apart from hospitalisations and deaths, influenza has significant economic consequences. The current egg-based inactivated influenza vaccines have several limitations: annual vaccination, high production costs, and cannot respond adequately to meet the demand during pandemics.

**Methods:**

We used a modified CHNRI methodology for setting priorities in health research investments. This was done in two stages. In Stage I, we systematically reviewed the literature related to emerging cross-protective vaccines against influenza relevant to several criteria of interest: answerability; cost of development, production and implementation; efficacy and effectiveness; deliverability, affordability and sustainability; maximum potential impact on disease burden reduction; acceptability to the end users and health workers; and effect on equity. In Stage II, we conducted an expert opinion exercise by inviting 20 experts (leading basic scientists, international public health researchers, international policy makers and representatives of pharmaceutical companies). They answered questions from the CHNRI framework and their “collective optimism” towards each criterion was documented on a scale from 0 to 100%.

**Results:**

The experts expressed very high level of optimism for deliverability, impact on equity, and acceptability to health workers and end users. However, they expressed concerns over the criteria of answerability, low development cost, low product cost, low implementation cost, affordability and, to a lesser extent sustainability. In addition they felt that the vaccine would have higher efficacy and impact on disease burden reduction on overall influenza-associated disease rather than specifically influenza-associated pneumonia.

**Conclusion:**

Although the landscape of emerging influenza vaccines shows several promising candidates, it is unlikely that the advancements in the newer vaccine technologies will be able to progress through to large scale production in the near future. The combined effects of continued investments in researching new vaccines and improvements of available vaccines will hopefully shorten the time needed to the development of an effective seasonal and pandemic influenza vaccine suitable for large scale production.

## Background

Globally, acute lower respiratory infections (ALRI) are a leading cause of morbidity and mortality in young children [1,2]. Respiratory viruses are commonly associated with ALRI episodes in young children [3]. Studies in the past decade suggested that the burden of disease due to hospital admissions for influenza associated ALRI in young and very young children is substantial [4,5]. Influenza is the second most commonly identified pathogen in children with ALRI and resulted in about 20 million new episodes of influenza-associated ALRI and 1 million hospitalisations in children aged below 5 years in the year 2008. Ninety six percent of these episodes were in developing countries. An estimated 28,000 to 111,500 children younger than 5 years of age died from influenza- associated ALRI in 2008, with 99% of these deaths occurring in developing countries. [6,7]. Apart from hospitalisations and deaths, influenza has significant economic consequences on families, healthcare services and society [4,8]. Therefore, governments may see broader value in using an influenza vaccine for example to avoid loss of work-days as well as reducing medical visits and hospital care episodes.

There are about 1.2 billion people at high risk for severe influenza outcomes who need an effective vaccine of which 385 million are over 65 years of age, 140 million are children under the age of five years, and 700 million are with an underlying chronic medical condition [9].

Annual vaccination remains the most effective way to significantly decrease the spread and subsequent mortality and morbidity associated with influenza viruses. However, the ability of the current egg-based inactivated vaccines to successfully provide long-term immunity is limited by *antigenic drift* (minor mutations causing small changes in the haemagglutinin gene); or the rarer *antigenic shift* (genetic re-assortment between animal and human influenza viruses; or a direct jump from animal species to humans of a virus that has acquired the ability to easily spread from human-to-human). Every year, the seasonal influenza vaccine is reformulated to match the circulating strains of the virus. Moreover, the production of egg-based vaccines are time and resource intensive and are unlikely to provide an adequate response during pandemics [10]. The poor uptake of the seasonal influenza vaccine (especially in high influenza burden developing countries) is linked to the limited production capacity in low resource settings and ultimately impairs the much needed surge in influenza vaccine production capacity during pandemics. The development of a novel influenza vaccine providing long term cross-protection has remained a scientific challenge. We aimed to review the existing literature, outlining the progress of the emerging vaccines against influenza at all stages of development; present the evidence regarding key issues surrounding these products and assess the level of collective optimism of international experts over its priority status for receiving investment support. The paper is presented as part of a series of papers addressing emerging vaccines and other interventions against pneumonia [11-16].

## Methods

We used a modified Child Health and Nutrition Research Initiative (CHNRI) methodology for setting priorities in health research investments. The original CHNRI methodology has been described in great detail [17-21] and implemented in a variety of settings [22-27]. The modification has been described in detail elsewhere [28] but is summarized below.

### CHNRI exercise – stage I: Identification and selection of studies

**Figure 1:**
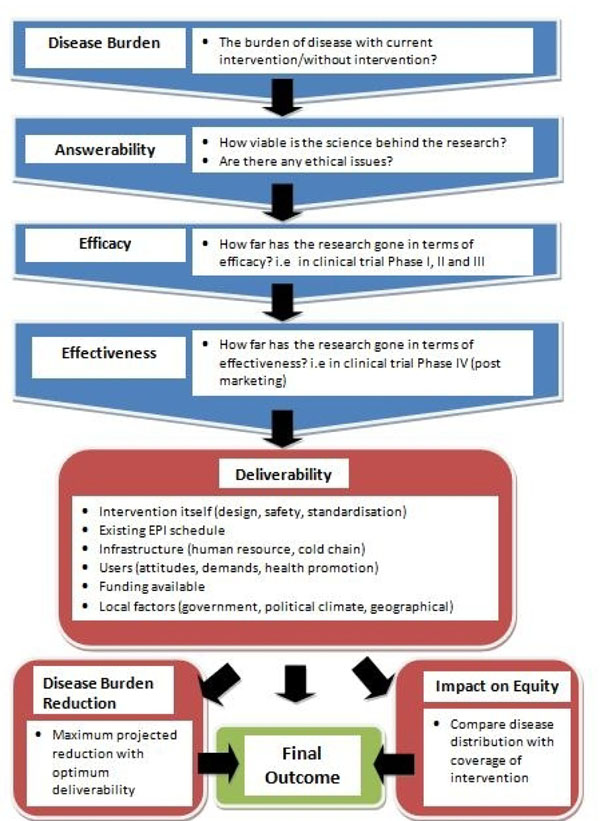
**A summary of Stage I of the CHNRI process of evaluation of an emerging intervention (a systematic review of the key CHNRI criteria)** CHNRI- Child Health and Nutrition Research Initiative

We conducted a systematic literature review using the following criteria: answerability, cost of development, cost of product, cost of implementation, efficacy and effectiveness, deliverability, affordability, sustainability, maximum potential impact on disease burden reduction, acceptability to health workers, acceptability to end users and equity [28] (Figure 1). The following search terms: influenza virus, vaccination, immunization, infants, and children were used. The search was limited to Ovid MEDLINE, Embase, Global Health, Web of Science, LILACS, IndMed, and grey literature (SIGLE) databases from July 2007 to June 2009 (updated in May 2012). A large part of the review involved searching the websites of individual pharmaceutical companies for details of research, including clinical trials, or product updates on vaccines in development. Relevant experts were also contacted for information regarding the various influenza vaccines under development. This was supplemented with hand searching of online journals and scanning of reference lists of identified citations. A total of 8021 articles were identified initially of which 81 articles were found suitable for full-text review. The inclusion and exclusion criteria are outlined in Table 1.

**Table 1:** Details of eligibility criteria used for screening the studies

Inclusion criteria	Exclusion Criteria
**- Included research into influenza vaccine, or other vaccine that may bear resemblance to future influenza vaccination programs**	- Influenza vaccine candidate was not a focus of the paper
**- Vaccine research was targeted at children under 5 years**	- Vaccines targeted at the elderly
**- Gave an indication of answerability, efficacy, effectiveness, deliverability, disease burden reduction or impact on equity of a vaccine**	- Papers not directly relating to vaccine development and its impact

### CHNRI exercise – stage II: An expert opinion exercise

**Figure 2:**
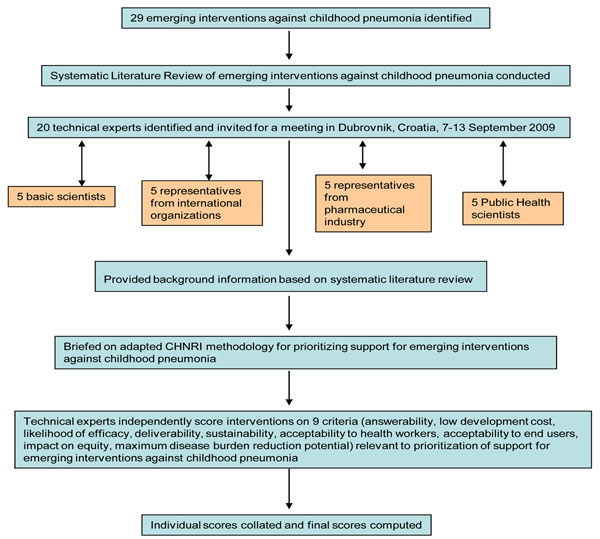
**A summary of Stage II of the CHNRI process of evaluation of an emerging intervention (an expert opinion exercise using the 9 CHNRI criteria)** CHNRI- Child Health and Nutrition Research Initiative

We shared the initial review of the literature (as background material) with 20 experts prior to the meeting. The list of chosen experts included five leading basic scientists, five international public health researchers, five international policy makers and five representatives of pharmaceutical companies which manufactured influenza vaccines. We initially offered participation to the 20 experts with the greatest impact of publications in their area of expertise over the past 5 years (for basic researchers and international public health researchers), or for being affiliated to the largest pharmaceutical companies. The policy makers and industry representatives accepted our invitation on the condition of anonymity, due to the sensitive nature of their involvement in such exercises. About half of the experts were either affiliated to institutions in developing countries or had previous experience of working in developing country settings. The experts met during September 7-13, 2009 in Dubrovnik, Croatia, to conduct the 2nd stage of CHNRI expert opinion exercise. The process of second-stage CHNRI is shown in Figure 2. The literature review on emerging interventions against childhood pneumonia (CHNRI stage I) were presented formally (using power point slides) using a structured format – the aforementioned CHNRI. After the evidence on a particular criteria (e.g. answerability) was presented, all invited experts discussed the evidence provided; the discussions were facilitated by IR and HC. The experts then independently answered questions from CHNRI framework (Additional file 1) following published guidelines [17-21].

## Results

We identified 40 articles in June 2009 (updated to 80 articles and product monographs in May 2012) for inclusion. We have presented the updated review in this paper. Currently 101 different influenza vaccines are in various stages of development, of which 78 are yet to enter Phase III clinical trials [29].

### Answerabilty - Is the science behind the research viable?

#### Adjuvanted egg-based inactivated vaccines (EBIV)

**Figure 3:**
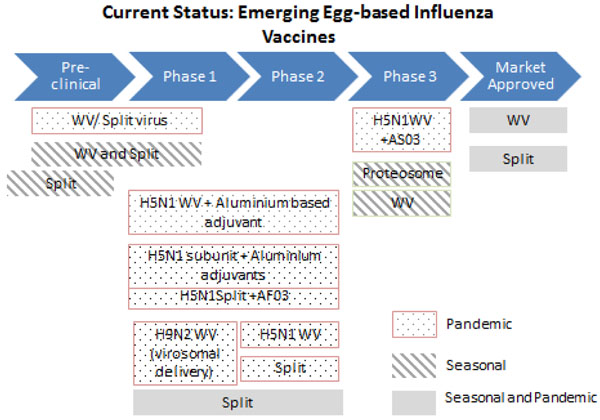
**The current status of the research into emerging egg-based influenza vaccines** WV- Inactivated Whole Virion

Adjuvanted vaccines (Figure 3) have been shown to be antigen sparing and more immunogenic compared to non-adjuvanted vaccines, and may allow increased production capacity to meet global demand [30-32].

#### Cell-cultured inactivated vaccines (CCIV)

**Figure 4:**
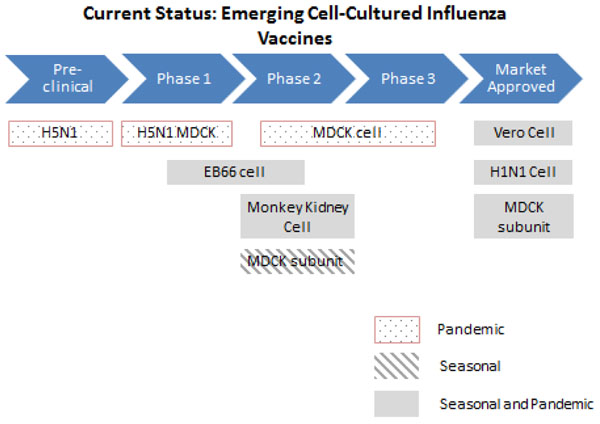
**The current status of the research into emerging cell-cultured influenza vaccines** MDCK- Madin-Darby Canine Kidney cells

CCIV (Figure 4) have been demonstrated to be equally well tolerated and show potentially greater flexibility of supply during periods of high demand compared to EBIV [33-35]. Madin-Darby Canine Kidney (MDCK) cells and Vero cells have been researched extensively and candidate vaccines- Optaflu (Novartis) and Cevapan (Baxter) are well tolerated and have gained regulatory approval in the EU [36,37]. Newer vaccines have also shown great promise during clinical trials. Although CCIV addresses many of the current limitations faced by EBIV, their production capacity is largely dependent on individual virus strains as some replicate better than others in mammalian cells. This is also a relatively new technology and requires more sophisticated equipment such as a fermenter-based cell culture either using suspension cells or a micro-carrier-based culture [38].

#### Live-attenuated influenza vaccines (LAIV)

**Figure 5:**
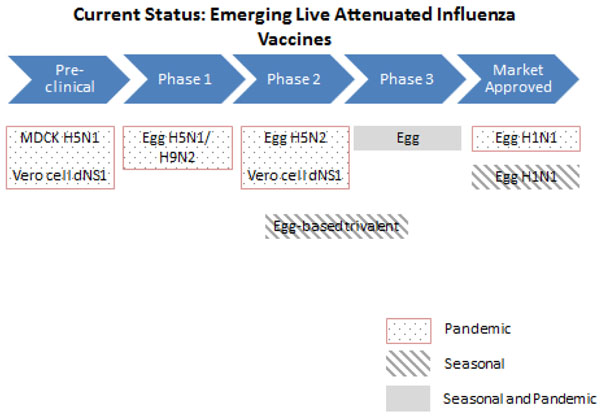
**The current status of the research into emerging live attenuated influenza vaccines** MDCK- Madin-Darby Canine Kidney cells

Current market-approved LAIVs are produced using the egg-based method (Figure 5) and therefore share the same advantages and limitations as EBIV. Drug companies are currently researching on developing LAIVs using cell-based technology. These have been shown to be safe and sufficient to produce a protective immune response in adult humans during Phase I and Phase II clinical trials and therefore might be an effective alternative to conventional EBIV [39-43].

#### Recombinant (VLP) vaccines

In the case of recombinant vaccines, the answerability would depend on the type of virus-like particles (VLP) used. Similar to CCIVs, these vaccines can be rapidly produced in large quantities while avoiding the use of eggs. Animal studies show that they are able to induce satisfactory immune response that correlates to protection [44-49].

**Figure 6:**
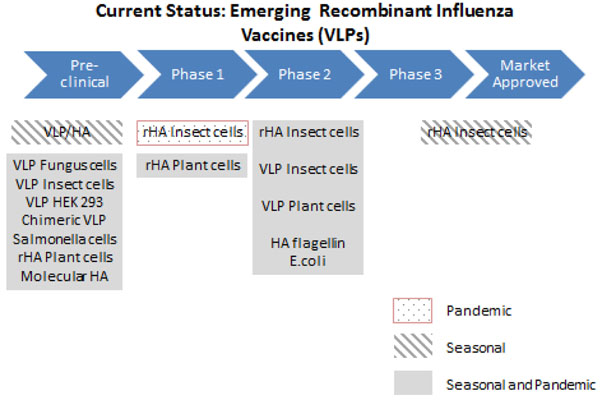
**The current status of the research into recombinant influenza vaccines** VLP- influenza virus like particles; HA- haemagglutinin; rHA- recombinant haemagglutinin

There are 19 companies currently developing different types of recombinant vaccines, indicating that there is great promise in the technology (Figure 6). Protein Sciences’ insect cell vaccines have shown the most progress (currently in Phase II trials) and have demonstrated a degree of cross protection against both influenza A(H1N1) and A(H3N2) strains [50-55]. Vaccine manufacturers remain optimistic that recombinant vaccines shall be able to meet the demands during pandemics. However, further research is required to evaluate the answerability of this technology.

#### Universal/Vector-based/DNA vaccines

**Figure 7:**
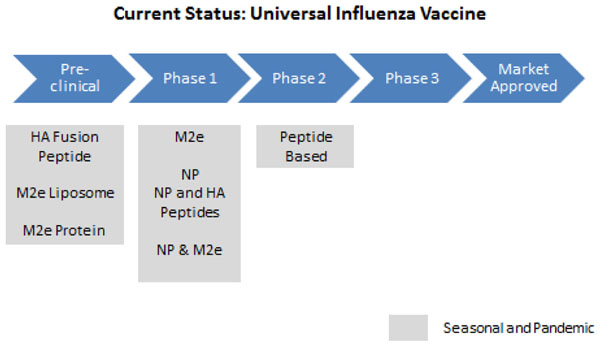
**The current status of the research into universal influenza vaccines** NP- novel peptide; HA- haemagglutinin

**Figure 8:**
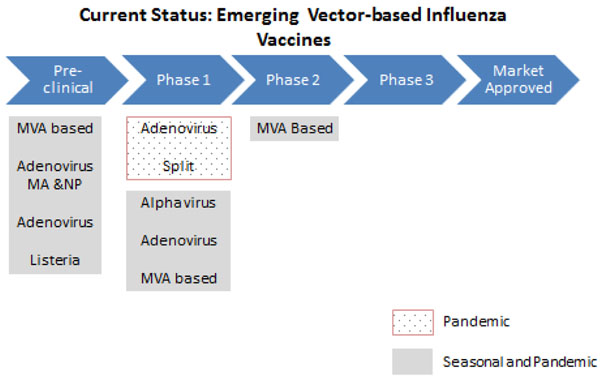
**The current status of the research into vector-based influenza vaccines** MVA- modified vaccinia virus Ankara; MA- multi-antigen; NP- nucleoprotein

**Figure 9:**
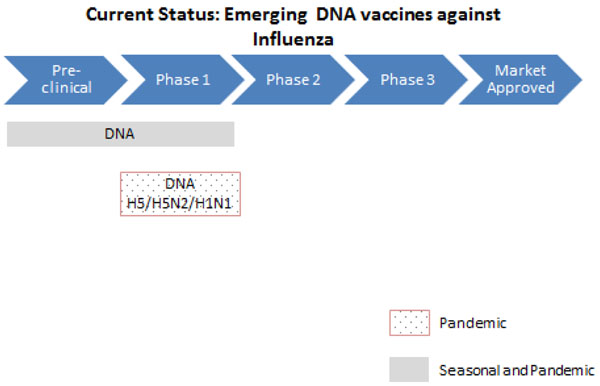
The current status of the research into DNA vaccines against influenza

Most of these vaccines are currently in pre-clinical and Phase I clinical trials (Figure 7, 8, 9). Therefore, more research is needed to evaluate the feasibility of these vaccines. Preliminary trials show that these vaccines are able to provide broad protective immunity across different influenza virus strains and are safe and well tolerated in animal and human studies [56-73].

**Figure 10:**
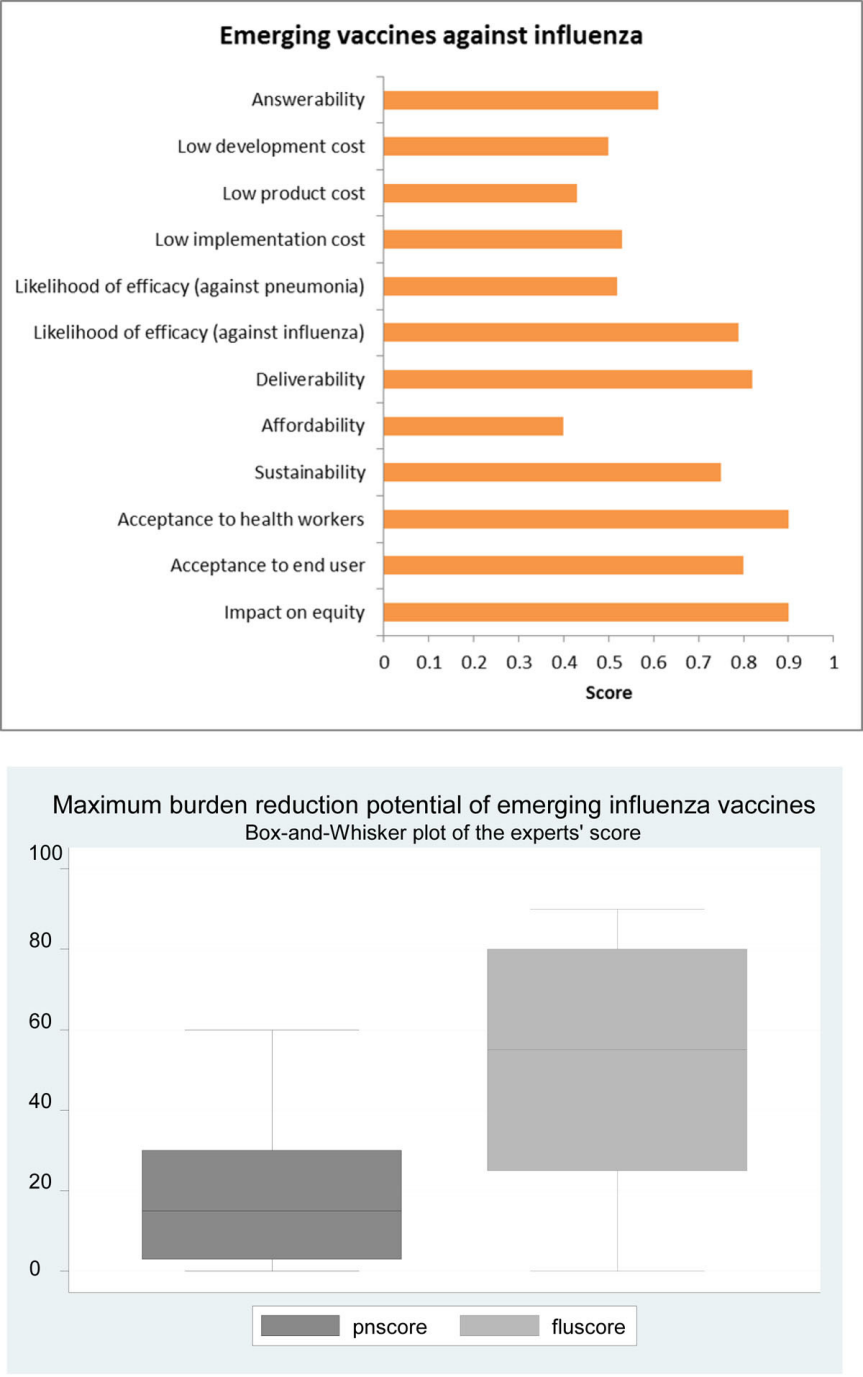
**The results of Stage II CHNRI process – an expert opinion exercise assessing the potential usefulness of investment in emerging influenza vaccines** CHNRI- Child Health and Nutrition Research Initiative

Based on this evidence, the panel of experts expressed concern over the ability of emerging cross-protective influenza vaccines to satisfy the criterion of answerability (score 61%) (Figure 10).

### Efficacy - the impact of the vaccines under ideal conditions

A recent Cochrane review reported that LAIVs have a good relative efficacy – 80 (95% CI 68 to 87) percent in children aged more than 2 years [74]. In comparison, inactivated vaccines have a relatively lower efficacy – 59 (95% CI 41 to 71) percent in this age group [75,76]. In a recent study, Johansson and colleagues demonstrated that viral co-infections increase the severity and duration of hospitalisation in patients with bacterial pneumonia [83]. For children aged below 2 years, although they reduce the risk for influenza by about a half, they are not significantly more efficacious than a placebo.

#### Adjuvanted egg-based inactivated vaccines

On-going phase II and III clinical trials studying the immunogenicity of adjuvanted vaccines have reported higher immune response compared to the non-adjuvanted formulation [30].

#### Cell-cultured inactivated vaccines

There are limited published data regarding the efficacy of newer CCIVs currently in development. Market approved CCIVs- Optaflu (Novartis) and Celvapan (Baxter) have both demonstrated adequate immunogenicity in large scale human studies and no serious adverse effects (SAEs) were reported [36,37]. They have both been approved by the European Medicines Agency (EMEA) in 2009. Both are undergoing phase III trials in the US. Bharat Biotech’s HNVAC has been tested in one of the largest phase I, II, and III clinical trials in India and has been proven immunogenic and well tolerated [77].

#### Live-attenuated influenza vaccines (LAIV)

LAIVs are currently licenced for use in in healthy children and adults between 2- 59 years old in Russia, India and USA [78]. A meta-analyses comparing the immunogenicity of intranasally administered LAIV with egg-based TIV in children (6 months to 17 years old) demonstrated the superior efficacy of the LAIV compared to TIV [79].

#### Recombinant (VLP) vaccines

VLP technology is relatively new and there are limited published data on their efficacy. Protein Science Corporation is developing seasonal (Flublok) and pandemic (Panblok) influenza vaccines which have shown favourable immunogenicity and tolerability during Phase I and II clinical trials [50,52,54]. Novavax’s H1N1 VLP vaccine was well tolerated and immunogenic in a phase II clinical trial carried out in more than 4000 subjects in Mexico [55]. Medicago have also announced promising Phase I trial results from the company’s plant cell based H1 VLP and H5 VLP vaccines and they intend to proceed with Phase IIa trial in the US [80].

#### Universal vaccines

Limited data are available regarding the efficacy of universal vaccines. Merck, Generex and Sanofi Pasteur have currently halted their clinical trials. Phase I data reported in February 2011 have found Dynavax’s universal candidate vaccine (N8295) to be safe and generally well tolerated alone or combined with H5N1 vaccine [58]. VaxInnate recently reported that its candidate vaccine, Vax102, safely produces an immune response in humans that should be protective against all strains of influenza A [59]. Biondvax’s Multimeric-001 universal vaccine has undergone phase IIa trials and was shown to successfully activate immune responses against the vaccine virus itself as well as the wild against Influenza A and B virus strains. The trial also demonstrated a higher antibody titre when co-administered with 50% TIV dose compared to TIV alone [63].

#### Vector-based vaccines

These vaccine candidates are in early trial stages, therefore there are limited data regarding their efficacy. Results from Erasmus, Emergent and CureLab’s preclinical trials demonstrate that the vaccines induce strong immune response to pandemic influenza virus antigens in healthy mice [60-62,64,65]. Results from AlphaVax Inc’s Phase I clinical trial in 2007 showed good immune response in volunteers, which persisted for four months [67]. Vaxin’s adenovirus-vectored vaccine and PaxVax’s PXVX- 0103 showed similar results in Phase I trials [68]. VaxArt Inc’s oral influenza vaccine has demonstrated adequate antibody response in animal subjects and is currently undergoing Phase I trials [69]. The Jenner Institute’s MVA-NP+M1 vaccine has already successfully progressed through a Phase I and Phase IIa study in human subjects and is being researched in combination with traditional TIV [70].

#### DNA vaccines

These vaccine candidates are in early phases of clinical trials. In preclinical animal studies and Phase I human trials, Inovio’s DNA vaccines demonstrated great potential of inducing immune response in healthy mice when delivered with their proprietary electroporation DNA delivery technique [72]. Data from Pfizer’s (previously PowderMed) DNA vaccination showed that the vaccine was well tolerated and induced sufficient antibody response to the influenza A/H3 Panama/2007/99 virus strain it was challenged with [81].

Based on this evidence, the experts were of the opinion that the likelihood of efficacy of these emerging cross-protective vaccines against influenza was high (score 79%) but was rather low against pneumonia (score 52%) (Figure 10).

### Effectiveness - maximum burden reduction potential

In a recent meta-analysis, Jefferson and colleagues have demonstrated that live attenuated vaccines have an overall effectiveness of 33% (RR in vaccinees 0.67; 95% CI 0.62 to 0.72), while inactivated vaccines have an overall effectiveness of 36% (RR in vaccinees 0.64; 95% CI 0.54 to 0.76) [74]. The authors could not find any evidence for children aged less than two years.

Immunisation with PCV7 has been shown to prevent hospitalisation for pneumonia in children with seasonal influenza – vaccine efficacy 45 (95% CI 14 to 64) percent [82]. In a recent study, Johansson and colleagues demonstrated that viral co-infection increase the severity and duration of hospitalisation in patients with bacterial pneumonia [83]. Thus, there is an unexplored potential of utilizing influenza vaccines to reduce the disease burden of childhood pneumonia [84]. A universal influenza vaccine that can cover all the virus subtypes, and provide effective and long lasting protection, might have a large impact on reducing the burden of childhood pneumonia.

The experts felt that on the criterion of the maximum potential impact on disease burden, the impact of a cross-protective vaccine would be greater on influenza-related mortality than overall pneumonia mortality- the median potential effectiveness of the vaccines in reduction of overall pneumonia was 13%; (interquartile range 3-30% and min. 0%, max 60%) compared to 60% for influenza-related mortality (interquartile range 25-80% and min. 0%, max 90%) (Figure 10).

### Deliverability, affordability and sustainability

This criterion takes into account the level of difficulty in delivering a novel influenza vaccine, the infrastructure and other resources available to implement the intervention and also government capacity and partnership requirements for achieving near-universal coverage with this new intervention.

#### Egg-based inactivated vaccines

The egg-based technology has been used for over 60 years and has proven to be an effective mode of production. However, its sustainability is dependent heavily on the supply of eggs and the estimated time required to establish a large scale plant is 4 years [78]. The technology, although established, is complex and requires advanced equipment which may not be available in many developing countries and therefore may not be sufficient to provide a comprehensive response to a pandemic outbreak [10].

#### Cell-cultured inactivated vaccines

The deliverability of CCIV has been compared extensively with that of EBIV and has shown to have an advantage in terms of production capacities and safety. The technology may also be used to develop other types of vaccines. However, it is a relatively new system with a variable yield of viral titres depending on the influenza virus strains [33,34]. Therefore, more comprehensive studies are required to evaluate the long term sustainability of this technology and its potential to preserve global demands.

#### LAIV

The sustainability of current LAIV is limited by its dependence on egg supply. The new cell-derived vaccine may be able to overcome this and improve the long term sustainability of LAIV. The aim is that it would be highly scalable with low manufacturing cost and free from animal or microbial contaminants [40,41].

#### Recombinant/Universal/ Vector-based/DNA vaccines

The specific deliverability and sustainability of these vaccine technologies are unknown as they are still in early trial stages. However, it is clear that they would require advanced and highly individualised equipment and processing plants which could take years to develop and implement. Although the drug companies involved in their production are positive regarding the sustainability of the vaccines, it is still too early to predict their long term results.

Should a candidate vaccine prove efficacious against pneumonia, future studies should assess their suitability for integration into an Expanded Programme of Immunisation (EPI) schedule. If the aim is to reduce pneumonia morbidity and mortality then vaccine administration should be aimed at children aged below 2 years [84], provided that they are sufficiently immunogenic and protective. The administration, transportation and storage should also complement those of the other EPI vaccines. [85]. The five main areas of current research to simplify administration are jet injection, intranasal spray, pulmonary inhalation of aerosols, oral ingestion and cutaneous administration (e.g. mechanical disruption of the stratum corneum, coated microtines, hollow micro-needles, dissolving micro-needles) [29,86]. The experts were optimistic (score over 80%) about the ability of the vaccine to satisfy the criteria of deliverability (Figure 10).

Various funding mechanisms, such as the International Finance Facility for Immunisation (IFFIm), the GAVI Alliance and Advance Market Commitment (AMC) for pneumococcal conjugate vaccines etc. exist for introduction and procurement of specific EPI vaccines to the least developed countries. The creation of any additional fund for influenza vaccines, unless it provides broad-spectrum and long lasting protection against constantly mutating viruses, is a challenge. The panel was also not optimistic (score about 50% and 40% respectively) about the ability to develop novel influenza vaccines at a low development cost and low production cost respectively– and hence were unsure whether vaccine prices could be kept low (Figure 10). The main argument for high costs associated with influenza vaccines is typically based on the requirement of annual reformulation and vaccination. Some experts pointed out that the price of a vaccine in a given country is often determined by negotiations between a government and an industry. Industry representatives explained that there were various elements to define a price: total volume produced and sustainability and predictability of demand. UNICEF uses a range of prices for developing country procurement. It was noted that only a limited number of vaccine manufacturers are interested in entering into UN and/or PAHO tenders and that vaccine price for private use may be marked up by a wholesaler and is influenced by intermediate sales and taxes which makes predicting the cost of both existing and emerging vaccines complex.

Current deliverability of the trivalent vaccine is unable to meet the enormous needs (see background) for several reasons. First, due to constant change in viral strains, there is a need for annual reformulation. Second, although vaccines stored in bulk are stable and could be used for stock-pile, no data on the exact shelf-life of the vaccines currently exist. Third, vaccines with virus types that are not optimally matched to wild-type virus have relatively lower efficacy than those with homologous strains [87], and thus are generally inadequate for subsequent seasons. Finally, although egg-based vaccine production is still the most common production method, it is not efficient, requiring 20 – 23 weeks for new vaccine production, as opposed to recombinant vaccines, which potentially could be produced in 8 – 12 weeks [88,89]. If Northern Hemisphere manufacturers based production on global rather than regional demand this would lead to a greater and most constant level of demand which would encourage facilities to increase vaccine production throughout the year. Furthermore, local manufacturing capacity in developing countries could be built up, which would provide surge potential for increased demand in times of pandemic or even greater seasonal uptake. Overall, there was some concern that even if the prices were kept low in the initial phases by support from GAVI Alliance and other agencies, high coverage with a novel cross-protective influenza vaccine might not be sustainable (score 75%) (Figure 10).

### Acceptability

Over the last five years, there has been an increase in the acceptability of influenza vaccines amongst both the healthcare workers and end-users. First, there has been significant expansion in global influenza vaccine manufacturing capacity, with seasonal vaccine production increasing from 350 million doses in 2006 to around 900 million doses in 2009 [90]. The WHO has been engaged in an influenza vaccine transfer technology transfer initiative to help create regionally based, independent and sustainable pandemic influenza vaccine production capacity in developing countries. [29]. Some countries with relatively low per capita GNIs such as Chile, Columbia, Panama and Mexico have achieved relatively high levels of distribution. Needle free delivery methods are likely to simplify vaccine administration and improve coverage in all settings [29]. The experts were highly optimistic (score over 80%) that the emerging cross-protective influenza vaccines would be acceptable to both the end-users and health workers (Figure 10).

### Equity

This considers predicted effects on poor, high burden populations within countries. [6] The score is high when the experts agree that the resultant impact will reduce health inequities between rich and poor social groups. The lowest social classes typically suffer from diminished intervention coverage, weaker health services and greater disease exposure. However, if the vaccine were to be made available to those at highest risk of disease (i.e. the most socio-economically deprived areas), there are likely to be substantial reductions both in age-specific mortality and in health inequalities [91]. This study highlights the importance of political will to ensure access of efficacious vaccine to the lower income, higher risk social sectors in order to achieve health equity. The experts were very optimistic (score over 80%) of the ability of the emerging cross-protective influenza vaccines to have a positive impact on equity (Figure 10).

## Discussion

This paper summarises the available evidence required for informing research and investment priority setting on cross-protective emerging influenza vaccines. While the experts expressed very high level of optimism for deliverability, impact on equity, and acceptability to health workers and end users, they expressed concerns over answerability, low development cost, low product cost, low implementation cost, affordability and, to a lesser extent sustainability. In addition they felt that the vaccine would have higher efficacy and impact on disease burden reduction on overall influenza-associated disease rather than specifically influenza-associated pneumonia. In some cases low scores on some criteria partly reflect lack of evidence. It is anticipated that in November 2012, based on the current evidence, the same panel of experts would score some of these criteria (especially the low scoring ones like answerability) quite differently.

This is the first time such an exercise has been attempted to make a structured assessment of emerging vaccines. The scores express the collective opinion of a panel of 20 experts. While there is always an element of uncertainty when predicting impact of interventions which do not exist, we feel that the results would be reproducible with another panel in a different setting. There might be some biases in the literature search as only we searched for studies published in English language. Inclusion of experts from five pharmaceutical companies manufacturing influenza vaccines and investing in research and development of emerging influenza vaccines may have also contributed to some bias. However, this is unlikely to have altered the final scores significantly.

Current research and developments are moving away from the egg-based technology and into cell cultured vaccines, which would effectively overcome the limitations of egg-based productions. Cell-culture production capacity can be scaled up quickly when needed and the product is free from animal contaminants. However, the cost of developing cell-cultured influenza vaccines is more expensive compared to egg-based production for quantities less than 25 million doses per year. The technology is also relatively new with new regulatory paths and therefore needs to be further refined in order to achieve the same level of success as EBIV.

The developmental success and limitations of LAIV are largely dependent on that of egg-based technology. Although still in clinical trial phases, cell-derived LAIV have shown promising results and may be able to replace the conventional egg-based LAIV in the near future. LAIV are administered intranasally and provides an exciting platform for the development of newer ‘friendly use’ vaccine delivery methods.

Currently, the development of recombinant vaccines using VLPs is one of the main focuses of the influenza vaccine industry. The very fact that numerous drug companies are currently researching this technology shows that there is great confidence in the efficacy and deliverability of these vaccines. VLP-produced vaccines are quicker to manufacture and potentially cheaper than currently available influenza vaccines, making these the forerunner in the race to develop a new vaccine platform to overcome the threat of a pandemic outbreak. However, questions remain as to whether the technology will be suitable for large scale production to overcome the production constraint of current vaccine technologies.

The development of a universal influenza vaccine has long been the ultimate goal of the industry. However, the feasibility of developing such a vaccine remains unclear as participating drug companies remain stagnant in their clinical trials. If successfully produced, the vaccine will have the potential to offer recipients long term and cross-protective immunisation against different virus strains. However, the scientific challenges notwithstanding, there are major economic and political impediments to the development of cross-protective influenza vaccine. A truly cross-protective vaccine that confers long-term protection (several years or more) would completely change the entire influenza vaccine market. The present market reflects many billions of dollars of investment and is highly profitable. Even today, additional egg-based vaccine capacity is being added by major manufacturers. A cross-protective influenza vaccine that confers long-term protection would represent a direct and major threat to the established business model. Such a novel vaccine would need to carry a very high price tag to compensate for the massive losses in income from the present annual vaccination model and the cost of developing entirely new production facilities (or retrofitting existing systems when feasible). Therefore, there is actually very little motivation for industry-sponsored research targeted at developing a universal vaccine. If such a vaccine is eventually developed, it will likely be the result of government and/or academic research.

Early studies of vector based vaccines have shown to increase the immunogenicity of traditional TIV and may be used in combination with current vaccines to provide a level of cross protection that is not characteristic of conventional influenza vaccines. Similarly, DNA vaccines are still in very premature stages of development and therefore it is difficult to predict its potential outcome. Although still in early stages, these vaccines show a great amount of potential in clinical trials in terms of their immunogenicity and production capacities.

Production and uptake of seasonal influenza vaccines is an integral part of pandemic influenza preparedness planning. In order to meet the demands for a surge in vaccine production during pandemics, technology transfer and establishment of regional centres for vaccine manufacture in resource poor settings have already been incorporated as part of the Global Action Plan (GAP) for influenza vaccines. However, integration of seasonal influenza vaccine into the EPI schedule (along with the vaccines against bacterial pneumonia) remains the key to decreasing childhood pneumonia morbidity and mortality.

## Conclusions

In summary, it is unlikely that the advancements in the newer vaccine technologies will be able to progress through to large scale production in the near future. Although arduous and time consuming, more clinical studies are needed to evaluate the viability and efficacy of these vaccines and their role in decreasing the global disease burden of influenza. The combined effects of continued investments in researching new vaccines and improvements of available vaccines will hopefully shorten the time needed to the development of an effective seasonal and pandemic influenza vaccine suitable for large scale production.

## Competing interests

SSJ is employed by Serum Institute of India Ltd. which manufactures the Live Attenuated Influenza Vaccine in India. WAB has received funding from the Bill & Melinda Gates Foundation for vaccine-related work in connection with childhood pneumonia; donation of vaccine from Sanofi-Pasteur for a vaccine trial against early childhood pneumonia; and project funding from Sanofi-Pasteur for pneumococcal vaccine trials and a study in pneumococcal pneumonia disease burden in young children; however, no grants or honoraria were received for work included in this study. HN, ESML, ACS, ET, LZ, TH, IR and HC declare that they have no competing interests.

## Authors’ contributions

HN supervised the literature review, participated in the design of the study, data collection, data analysis, data interpretation and prepared the initial draft of the manuscript. ESML conducted the literature review and contributed to preparation of the initial draft of the manuscript. ACS participated in the literature review and contributed to data collection. ET participated in design of the study, data collection, statistical analysis and data interpretation. LZ participated in the design of the study, data collection, data collection and data interpretation. SSJ and WAB contributed to data interpretation and critical review of the manuscript. IR and HC conceived the study, participated in data collection, data interpretation, and critically reviewed drafts of the manuscript. All authors read and approved the final manuscript.

## Supplementary Material

Additional file 1 Questions used in the Phase II CHNRI process Click here for file

## Declarations

The publication costs for this supplement were funded by a grant from the Bill & Melinda Gates Foundation to the US Fund for UNICEF (grant 43386 to "Promote evidence-based decision making in designing maternal, neonatal, and child health interventions in low- and middle-income countries”). The Supplement Editor is the principal investigator and lead in the development of the Lives Saved Tool (LiST), supported by grant 43386. He declares that he has no competing interests.

This article has been published as part of *BMC Public Health* Volume 13 Supplement 3, 2013: The Lives Saved Tool in 2013: new capabilities and applications. The full contents of the supplement are available online at http://www.biomedcentral.com/bmcpublichealth/supplements/13/S3.

## References

[B1] Rudan I Boschi-Pinto C Biloglav Z Mulholland K Campbell H Epidemiology and etiology of childhood pneumonia Bull World Health Organ 2008 86 5 408 416 10.2471/BLT.07.048769 18545744 PMC2647437

[B2] Liu L Johnson HL Cousens S Perin J Scott S Lawn JE Rudan I Campbell H Cibulskis R Li M Global, regional, and national causes of child mortality: an updated systematic analysis for 2010 with time trends since 2000 Lancet 2012 379 9832 2151 2161 10.1016/S0140-6736(12)60560-1 22579125

[B3] Ruuskanen O Lahti E Jennings LC Murdoch DR Viral pneumonia Lancet 2011 377 9773 1264 1275 10.1016/S0140-6736(10)61459-6 21435708 PMC7138033

[B4] Neuzil KM Zhu YW Griffin MR Edwards KM Thompson JM Tollefson SJ Wright PF Burden of interpandemic influenza in children younger than 5 years: a 25-year prospective study J Infect Dis 2002 185 2 147 152 10.1086/338363 11807687

[B5] Poehling KA Edwards KM Weinberg GA Szilagyi P Staat MA Iwane MK Bridges CB Grijalva CG Zhu YW Bernstein DI The underrecognized burden of influenza in young children N Engl J Med 2006 355 1 31 40 10.1056/NEJMoa054869 16822994

[B6] Nair H Brooks WA Katz M Roca A Berkley JA Madhi SA Simmerman JM Gordon A Sato M Howie S Global burden of respiratory infections due to seasonal influenza in young children: a systematic review and meta-analysis Lancet 2011 378 9807 1917 1930 10.1016/S0140-6736(11)61051-9 22078723

[B7] Brooks WA Goswami D Rahman M Nahar K Fry AM Balish A Iftekharuddin N Azim T X X Klimov A Influenza is a major contributor to childhood pneumonia in a tropical developing country Pediatr Infect Dis J 2010 29 3 216 221 10.1097/INF.0b013e3181bc23fd 20190613

[B8] Principi N Esposito S Marchisio P Gasparini R Crovari P Socioeconomic impact of influenza on healthy children and their families Pediatr Infect Dis J 2003 22 10 Suppl S207 210 14551476 10.1097/01.inf.0000092188.48726.e4

[B9] Initiative for Vaccines Research Team Department of Immunization VaB State of the art of vaccine research and development 2005 Geneva: World Health Organization 14 17

[B10] Kieny MP Costa A Hombach J Carrasco P Pervikov Y Salisbury D Greco M Gust I LaForce M Franco-Paredes C A global pandemic influenza vaccine action plan Vaccine 2006 24 40-41 6367 6370 10.1016/j.vaccine.2006.07.021 17240560

[B11] Higginson D Theodoratou E Nair H Huda T Zgaga L Jadhav SS Omer SB Rudan I Campbell H An evaluation of respiratory administration of measles vaccine for prevention of acute lower respiratory infections in children BMC public health 2011 11 Suppl 3 S31 10.1186/1471-2458-11-S3-S31 21501450 PMC3231905

[B12] Nair H Verma VR Theodoratou E Zgaga L Huda T Simoes EA Wright PF Rudan I Campbell H An evaluation of the emerging interventions against Respiratory Syncytial Virus (RSV)-associated acute lower respiratory infections in children BMC public health 2011 11 Suppl 3 S30 10.1186/1471-2458-11-S3-S30 21501449 PMC3231904

[B13] Choudhuri D Huda T Theodoratou E Nair H Zgaga L Falconer R Luksic I Johnson HL Zhang JS El Arifeen S An evaluation of emerging vaccines for childhood meningococcal disease BMC public health 2011 11 Suppl 3 S29 10.1186/1471-2458-11-S3-S29 21501447 PMC3231902

[B14] Catto AG Zgaga L Theodoratou E Huda T Nair H El Arifeen S Rudan I Duke T Campbell H An evaluation of oxygen systems for treatment of childhood pneumonia BMC public health 2011 11 Suppl 3 S28 10.1186/1471-2458-11-S3-S28 21501446 PMC3231901

[B15] Huda T Nair H Theodoratou E Zgaga L Fattom A El Arifeen S Rubens C Campbell H Rudan I An evaluation of the emerging vaccines and immunotherapy against staphylococcal pneumonia in children BMC public health 2011 11 Suppl 3 S27 10.1186/1471-2458-11-S3-S27 21501445 PMC3239838

[B16] Webster J Theodoratou E Nair H Seong AC Zgaga L Huda T Johnson HL Madhi S Rubens C Zhang JS An evaluation of emerging vaccines for childhood pneumococcal pneumonia BMC public health 2011 11 Suppl 3 S26 10.1186/1471-2458-11-S3-S26 21501444 PMC3231900

[B17] Rudan I Gibson JL Ameratunga S El Arifeen S Bhutta ZA Black M Black RE Brown KH Campbell H Carneiro I Setting priorities in global child health research investments: guidelines for implementation of CHNRI method Croat Med J 2008 49 6 720 733 10.3325/cmj.2008.49.720 19090596 PMC2621022

[B18] Rudan I The complex challenge of setting priorities in health research investments Indian J Med Res 2009 129 4 351 353 19535827

[B19] Rudan I Chopra M Kapiriri L Gibson J Ann Lansang M Carneiro I Ameratunga S Tsai AC Chan KY Tomlinson M Setting priorities in global child health research investments: universal challenges and conceptual framework Croat Med J 2008 49 3 307 317 10.3325/cmj.2008.3.307 18581609 PMC2443616

[B20] Rudan I El Arifeen S Black RE Campbell H Childhood pneumonia and diarrhoea: setting our priorities right Lancet Infect Dis 2007 7 1 56 61 10.1016/S1473-3099(06)70687-9 17182344

[B21] Rudan I Gibson J Kapiriri L Lansang MA Hyder AA Lawn J Darmstadt GL Cousens S Bhutta ZA Brown KH Setting priorities in global child health research investments: assessment of principles and practice Croat Med J 2007 48 5 595 604 17948946 PMC2205967

[B22] Bahl R Martines J Ali N Bhan MK Carlo W Chan KY Darmstadt GL Hamer DH Lawn JE McMillan DD Research priorities to reduce global mortality from newborn infections by 2015 Pediatr Infect Dis J 2009 28 1 Suppl S43 48 19106763 10.1097/INF.0b013e31819588d7

[B23] Fontaine O Kosek M Bhatnagar S Boschi-Pinto C Chan KY Duggan C Martinez H Ribeiro H Rollins NC Salam MA Setting research priorities to reduce global mortality from childhood diarrhoea by 2015 PLoS Med 2009 6 3 e41 10.1371/journal.pmed.1000041 19278292 PMC2653551

[B24] Kapiriri L Tomlinson M Chopra M El Arifeen S Black RE Rudan I Setting priorities in global child health research investments: addressing values of stakeholders Croat Med J 2007 48 5 618 627 17948948 PMC2213572

[B25] Tomlinson M Chopra M Sanders D Bradshaw D Hendricks M Greenfield D Black RE El Arifeen S Rudan I Setting priorities in child health research investments for South Africa PLoS Med 2007 4 8 e259 10.1371/journal.pmed.0040259 17760497 PMC1952202

[B26] Tomlinson M Rudan I Saxena S Swartz L Tsai AC Patel V Setting priorities for global mental health research Bull World Health Organ 2009 87 6 438 446 10.2471/BLT.08.054353 19565122 PMC2686213

[B27] Tomlinson M Swartz L Officer A Chan KY Rudan I Saxena S Research priorities for health of people with disabilities: an expert opinion exercise Lancet 2009 374 9704 1857 1862 10.1016/S0140-6736(09)61910-3 19944866

[B28] Rudan I Theodoratou E Zgaga L Nair H Chan KY Tomlinson M Tsai AC Biloglav Z Huda T El Arifeen S Setting priorities for development of emerging interventions against childhood pneumonia, meningitis and influenza J Glob Health 2012 2 1 10304 23198129 10.7189/jogh.02.010304 PMC3484764

[B29] World Health Organisation Report of the second WHO Consultation on the Global Action Plan for Influenza Vaccines (GAP) 2011 Geneva, Switzerland: World Health Organisation

[B30] Waddington CS Walker WT Oeser C Reiner A John T Wilkins S Casey M Eccleston PE Allen RJ Okike I Safety and immunogenicity of AS03B adjuvanted split virion versus non-adjuvanted whole virion H1N1 influenza vaccine in UK children aged 6 months-12 years: open label, randomised, parallel group, multicentre study BMJ 2010 340 c2649 10.1136/bmj.c2649 20508026 PMC2877808

[B31] Carter NJ Plosker GL Prepandemic influenza vaccine H5N1 (split virion, inactivated, adjuvanted) [Prepandrix]: a review of its use as an active immunization against influenza A subtype H5N1 virus BioDrugs 2008 22 5 279 292 10.2165/00063030-200822050-00001 18778110

[B32] Leroux-Roels I Roman F Forgus S Maes C De Boever F Drame M Gillard P van der Most R Van Mechelen M Hanon E Priming with AS03 A-adjuvanted H5N1 influenza vaccine improves the kinetics, magnitude and durability of the immune response after a heterologous booster vaccination: an open non-randomised extension of a double-blind randomised primary study Vaccine 2010 28 3 849 857 10.1016/j.vaccine.2009.10.017 19835828

[B33] Frey S Vesikari T Szymczakiewicz-Multanowska A Lattanzi M Izu A Groth N Holmes S Clinical efficacy of cell culture-derived and egg-derived inactivated subunit influenza vaccines in healthy adults Clin Infect Dis 2010 51 9 997 1004 10.1086/656578 20868284

[B34] Szymczakiewicz-Multanowska A Groth N Bugarini R Lattanzi M Casula D Hilbert A Tsai T Podda A Safety and immunogenicity of a novel influenza subunit vaccine produced in mammalian cell culture J Infect Dis 2009 200 6 841 848 10.1086/605505 19673651

[B35] Vesikari T Block SL Guerra F Lattanzi M Holmes S Izu A Gaitatzis N Hilbert AK Groth N Immunogenicity, safety and reactogenicity of a mammalian cell-culture-derived influenza vaccine in healthy children and adolescents three to seventeen years of age Pediatr Infect Dis J 2012 31 5 494 500 10.1097/INF.0b013e31824bb179 22301476

[B36] Optaflu http://www.emea.europa.eu/docs/en_GB/document_library/EPAR_-_Summary_for_the_public/human/000758/WC500046952.pdf

[B37] Celvapan http://www.ema.europa.eu/docs/en_GB/document_library/EPAR_-_Summary_for_the_public/human/000982/WC500022671.pdf

[B38] World Health Organisation Initiative for Vaccine Research A review of production technologies for influenza virus vaccines, and their suitability for deployment in developing countries for influenza pandemic preparedness 2006 Geneva: World Health Organisation

[B39] Liu J S X Schwartz R Kemble G Use of MDCK cells for production of live attenuated influenza vaccine Vaccine 2009 27 46 6460 6463 10.1016/j.vaccine.2009.06.024 19559113

[B40] Zhou B Li Y Belser JA Pearce MB Schmolke M Subba AX Shi Z Zaki SR Blau DM Garcia-Sastre A NS-based live attenuated H1N1 pandemic vaccines protect mice and ferrets Vaccine 2010 28 50 8015 8025 10.1016/j.vaccine.2010.08.106 20934458 PMC2991506

[B41] George M Farooq M Dang T Cortes B Liu J Maranga L Production of cell culture (MDCK) derived live attenuated influenza vaccine (LAIV) in a fully disposable platform process Biotechnol Bioeng 2010 106 6 906 917 10.1002/bit.22753 20589670

[B42] Baskin CR Bielefeldt-Ohmann H Garcia-Sastre A Tumpey TM Van Hoeven N Carter VS Thomas MJ Proll S Solorzano A Billharz R Functional genomic and serological analysis of the protective immune response resulting from vaccination of macaques with an NS1-truncated influenza virus J Virol 2007 81 21 11817 11827 10.1128/JVI.00590-07 17715226 PMC2168783

[B43] Vincent AL Ma W Lager KM Janke BH Webby RJ Garcia-Sastre A Richt JA Efficacy of intranasal administration of a truncated NS1 modified live influenza virus vaccine in swine Vaccine 2007 25 47 7999 8009 10.1016/j.vaccine.2007.09.019 17933442 PMC2099695

[B44] Matassov D Cupo A Galarza JM A novel intranasal virus-like particle (VLP) vaccine designed to protect against the pandemic 1918 influenza A virus (H1N1) Viral Immunol 2007 20 3 441 452 10.1089/vim.2007.0027 17931114

[B45] Weldon WC Martin MP Zarnitsyn V Wang B Koutsonanos D Skountzou I Prausnitz MR Compans RW Microneedle vaccination with stabilized recombinant influenza virus hemagglutinin induces improved protective immunity Clin Vaccine Immunol 2011 18 4 647 654 10.1128/CVI.00435-10 21288996 PMC3122571

[B46] Iyer V Liyanage MR Shoji Y Chichester JA Jones RM Yusibov V Joshi SB Middaugh CR Formulation development of a plant-derived h1n1 influenza vaccine containing purified recombinant hemagglutinin antigen Hum Vaccin Immunother 2012 8 4 453 464 22370514 10.4161/hv.19106

[B47] Shoji Y Chichester JA Bi H Musiychuk K de la Rosa P Goldschmidt L Horsey A Ugulava N Palmer GA Mett V Plant-expressed HA as a seasonal influenza vaccine candidate Vaccine 2008 26 23 2930 2934 10.1016/j.vaccine.2008.03.045 18440103

[B48] Athmaram TN Saraswat S Santhosh SR Singh AK Suryanarayana WS Priya R Gopalan N Parida M Rao PV Vijayaraghavan R Yeast expressed recombinant Hemagglutinin protein of novel H1N1 elicits neutralising antibodies in rabbits and mice Virol J 2011 8 524 10.1186/1743-422X-8-524 22126628 PMC3251546

[B49] Liu G Tarbet B Song L Reiserova L Weaver B Chen Y Li H Hou F Liu X Parent J Immunogenicity and efficacy of flagellin-fused vaccine candidates targeting 2009 pandemic H1N1 influenza in mice PLoS One 2011 6 6 e20928 10.1371/journal.pone.0020928 21687743 PMC3110246

[B50] Baxter R Patriarca PA Ensor K Izikson R Goldenthal KL Cox MM Evaluation of the safety, reactogenicity and immunogenicity of FluBlok(R) trivalent recombinant baculovirus-expressed hemagglutinin influenza vaccine administered intramuscularly to healthy adults 50-64 years of age Vaccine 2011 29 12 2272 2278 10.1016/j.vaccine.2011.01.039 21277410

[B51] Cox MM Hashimoto Y A fast track influenza virus vaccine produced in insect cells J Invertebr Pathol 2011 107 Suppl S31 41 21784229 10.1016/j.jip.2011.05.003

[B52] Treanor JJ El Sahly H King J Graham I Izikson R Kohberger R Patriarca P Cox M Protective efficacy of a trivalent recombinant hemagglutinin protein vaccine (FluBlok(R)) against influenza in healthy adults: a randomized, placebo-controlled trial Vaccine 2011 29 44 7733 7739 10.1016/j.vaccine.2011.07.128 21835220

[B53] Khurana S Wu J Verma N Verma S Raghunandan R Manischewitz J King LR Kpamegan E Pincus S Smith G H5N1 virus-like particle vaccine elicits cross-reactive neutralizing antibodies that preferentially bind to the oligomeric form of influenza virus hemagglutinin in humans J Virol 2011 85 21 10945 10954 10.1128/JVI.05406-11 21865396 PMC3194932

[B54] King JC Jr. Cox MM Reisinger K Hedrick J Graham I Patriarca P Evaluation of the safety, reactogenicity and immunogenicity of FluBlok trivalent recombinant baculovirus-expressed hemagglutinin influenza vaccine administered intramuscularly to healthy children aged 6-59 months Vaccine 2009 27 47 6589 6594 10.1016/j.vaccine.2009.08.032 19716456

[B55] Lopez-Macias C Ferat-Osorio E Tenorio-Calvo A Isibasi A Talavera J Arteaga-Ruiz O Arriaga-Pizano L Hickman SP Allende M Lenhard K Safety and immunogenicity of a virus-like particle pandemic influenza A (H1N1) 2009 vaccine in a blinded, randomized, placebo-controlled trial of adults in Mexico Vaccine 2011 29 44 7826 7834 10.1016/j.vaccine.2011.07.099 21816199 PMC7126971

[B56] Stanekova Z Kiraly J Stropkovska A Mikuskova T Mucha V Kostolansky F Vareckova E Heterosubtypic protective immunity against influenza A virus induced by fusion peptide of the hemagglutinin in comparison to ectodomain of M2 protein Acta Virol 2011 55 1 61 67 10.4149/av_2011_01_61 21434706

[B57] Zhou D Wu TL Lasaro MO Latimer BP Parzych EM Bian A Li Y Li H Erikson J Xiang Z A universal influenza A vaccine based on adenovirus expressing matrix-2 ectodomain and nucleoprotein protects mice from lethal challenge Mol Ther 2010 18 12 2182 2189 10.1038/mt.2010.202 20877342 PMC2997593

[B58] Press release. Dynavax Reports New Phase 1a and Phase 1b Data for Universal Flu Vaccine Candidate http://investors.dynavax.com/releasedetail.cfm?ReleaseID=551606

[B59] VaxInnate’s Universal Flu Vaccine Candidate Shown Safe and Immunogenic in Phase I Clinical Study http://www.vaxinnate.com/pages/pressreleases/20081026_001.html

[B60] Kreijtz JH Suezer Y de Mutsert G van Amerongen G Schwantes A van den Brand JM Fouchier RA Lower J Osterhaus AD Sutter G MVA-based H5N1 vaccine affords cross-clade protection in mice against influenza A/H5N1 viruses at low doses and after single immunization PLoS One 2009 4 11 e7790 10.1371/journal.pone.0007790 19915662 PMC2771904

[B61] Kreijtz JH Suezer Y de Mutsert G van den Brand JM van Amerongen G Schnierle BS Kuiken T Fouchier RA Lower J Osterhaus AD Preclinical evaluation of a modified vaccinia virus Ankara (MVA)-based vaccine against influenza A/H5N1 viruses Vaccine 2009 27 45 6296 6299 10.1016/j.vaccine.2009.03.020 19840663

[B62] Rimmelzwaan GF Sutter G Candidate influenza vaccines based on recombinant modified vaccinia virus Ankara Expert Rev Vaccines 2009 8 4 447 454 10.1586/erv.09.4 19348560 PMC9709929

[B63] The Multimeric Universal Influenza Vaccine: BVX-M001 http://www.biondvax.com/128084/Product

[B64] Tang DC Zhang J Toro H Shi Z Van Kampen KR Adenovirus as a carrier for the development of influenza virus-free avian influenza vaccines Expert Rev Vaccines 2009 8 4 469 481 10.1586/erv.09.1 19348562 PMC2778197

[B65] Johnson PV Blair BM Zeller S Kotton CN Hohmann EL Attenuated Listeria monocytogenes vaccine vectors expressing influenza A nucleoprotein: preclinical evaluation and oral inoculation of volunteers Microbiol Immunol 2011 55 5 304 317 10.1111/j.1348-0421.2011.00322.x 21338384 PMC3082616

[B66] Holman DH Wang D Raja NU Luo M Moore KM Woraratanadharm J Mytle N Dong JY Multi-antigen vaccines based on complex adenovirus vectors induce protective immune responses against H5N1 avian influenza viruses Vaccine 2008 26 21 2627 2639 10.1016/j.vaccine.2008.02.053 18395306

[B67] AlphaVax announces results from initial testing of its H1N1 (swine) influenza vaccine http://www.redorbit.com/news/health/1722686/alphavax_announces_results_from_initial_testing_of_its_h1n1_swine/

[B68] Our avian influenza (H5N1) vaccine candidate http://paxvax.com/the-paxvax-solution/our-products

[B69] Vaxart Begins First Oral Vaccine Clinical Trial http://www.vaxart.com/files/VaxartPh1Trial.pdf

[B70] Vector Engineering http://www.jenner.ac.uk/vector-engineering

[B71] Jazayeri SD Ideris A Zakaria Z Shameli K Moeini H Omar AR Cytotoxicity and immunological responses following oral vaccination of nanoencapsulated avian influenza virus H5 DNA vaccine with green synthesis silver nanoparticles J Control Release 2012 161 1 116 123 10.1016/j.jconrel.2012.04.015 22549012

[B72] DNA / SnyCon w/ Electroporation Pipeline http://www.inovio.com/products/index.htm

[B73] NIAID Testing Candidate DNA Vaccine for 2009 H1N1 Influenza http://www.niaid.nih.gov/news/newsreleases/2009/Pages/CandidateDNAvaccine.aspx

[B74] Jefferson T Rivetti A Di Pietrantonj C Demicheli V Ferroni E Vaccines for preventing influenza in healthy children Cochrane Database Syst Rev 2012 8 CD004879 22895945 10.1002/14651858.CD004879.pub4 PMC6478137

[B75] Negri E Colombo C Giordano L Groth N Apolone G La Vecchia C Influenza vaccine in healthy children: a meta-analysis Vaccine 2005 23 22 2851 2861 10.1016/j.vaccine.2004.11.053 15780733

[B76] Zangwill KM Belshe RB Safety and efficacy of trivalent inactivated influenza vaccine in young children: a summary for the new era of routine vaccination Pediatr Infect Dis J 2004 23 3 189 197 10.1097/01.inf.0000116292.46143.d6 15014289

[B77] Kumar P Bharat Biotech launches first cell culture-based H1N1 vaccine in India BioSpectrum (Asia Edition) 2011 5 26 27

[B78] 7th WHO Meeting on Evaluation of Pandemic Influenza Vaccines in Clinical Trials http://www.who.int/vaccine_research/diseases/influenza/meeting_17_18Feb2011/en/index.html 10.1016/j.vaccine.2011.08.031 21856358

[B79] Rhorer J Ambrose CS Dickinson S Hamilton H Oleka NA Malinoski FJ Wittes J Efficacy of live attenuated influenza vaccine in children: A meta-analysis of nine randomized clinical trials Vaccine 2009 27 7 1101 1110 10.1016/j.vaccine.2008.11.093 19095024

[B80] Medicago Successfully Completes the Production of More Than Ten Million Doses of H1N1 VLP Influenza Vaccine in One Month http://www.medicago.com/English/news/News-Releases/News-ReleaseDetails/2012/Medicago-Successfully-Completes-the-Production-of-More-Than-Ten-Million-Doses-of-H1N1-VLP-Influenza-Vaccine-in-One-Month11302/default.aspx

[B81] Jones S Evans K McElwaine-Johnn H Sharpe M Oxford J Lambkin-Williams R Mant T Nolan A Zambon M Ellis J DNA vaccination protects against an influenza challenge in a double-blind randomised placebo-controlled phase 1b clinical trial Vaccine 2009 27 18 2506 2512 10.1016/j.vaccine.2009.02.061 19368793

[B82] Madhi SA Klugman KP A role for Streptococcus pneumoniae in virus-associated pneumonia Nature medicine 2004 10 8 811 813 10.1038/nm1077 15247911 PMC7095883

[B83] Johansson N Kalin M Hedlund J Clinical impact of combined viral and bacterial infection in patients with community-acquired pneumonia Scandinavian journal of infectious diseases 2011 43 8 609 615 10.3109/00365548.2011.570785 21466255

[B84] Brooks WA A four-stage strategy to reduce childhood pneumonia-related mortality by 2015 and beyond Vaccine 2009 27 5 619 623 10.1016/j.vaccine.2008.10.071 19010371

[B85] Mahoney RT Maynard JE The introduction of new vaccines into developing countries Vaccine 1999 17 7-8 646 652 10.1016/S0264-410X(98)00246-1 10067669

[B86] Alarcon JB Hartley AW Harvey NG Mikszta JA Preclinical evaluation of microneedle technology for intradermal delivery of influenza vaccines Clin Vaccine Immunol 2007 14 4 375 381 10.1128/CVI.00387-06 17329444 PMC1865614

[B87] Jefferson TO Rivetti D Di Pietrantonj C Rivetti A Demicheli V Vaccines for preventing influenza in healthy adults Cochrane Database Syst Rev 2007 2 CD001269 17443504 10.1002/14651858.CD001269.pub3

[B88] Penney CA Thomas DR Deen SS Walmsley AM Plant-made vaccines in support of the Millennium Development Goals Plant cell reports 2011 30 5 789 798 10.1007/s00299-010-0995-5 21243362 PMC3075396

[B89] Oliver Wyman Influenza Vaccine Strategies for Broad Global Access- Key Findings and Project Methodology 2007 PATH

[B90] Collin N de Radigues X Vaccine production capacity for seasonal and pandemic (H1N1) 2009 influenza Vaccine 2009 27 38 5184 5186 10.1016/j.vaccine.2009.06.034 19563891

[B91] Antunes JL Waldman EA Borrell C Paiva TM Effectiveness of influenza vaccination and its impact on health inequalities International journal of epidemiology 2007 36 6 1319 1326 10.1093/ije/dym208 17977871

